# The Role of Oxidative Stress and Natural Products in Maintaining Human Health

**DOI:** 10.3390/nu16091268

**Published:** 2024-04-25

**Authors:** Hui-Hui Xiao

**Affiliations:** 1 Research Center for Chinese Medicine Innovation, The Hong Kong Polytechnic University, Hong Kong, China; huihui.xiao@polyu.edu.hk; 2 Department of Food Science and Nutrition, The Hong Kong Polytechnic University, Hong Kong, China; 3 State Key Laboratory of Chinese Medicine and Molecular Pharmacology (Incubation), The Hong Kong Polytechnic University Shenzhen Research Institute, Shenzhen 518057, China

Since 1985, when oxidative stress was first defined as the oxidative damage caused to cells and organs, a large number of studies have shown that oxidative stress is a significant risk factor for various diseases, including tumors [1], cardiovascular [2] and cerebrovascular diseases [3], diabetes [4], and neurodegenerative diseases such as Alzheimer’s disease [5]. In recent years, there has been growing interest in the dual effects of oxidative stress on biological processes, which can be both beneficial and detrimental depending on the context and the degree of the stress [6]. It is involved in a multitude of biological processes, such as DNA damage and repair [7], cell growth, differentiation, apoptosis and metabolism [8], immune response [9] and inflammation [10].

Over the past three decades, the study of oxidative stress has gained significant momentum in the scientific community, with researchers exploring various strategies to counter its deleterious effects. Bioactive compounds composing daily consumed worldwide products considered as healthy such as coffee [11], tea [12], fruits, vegetables [13], herbals [14,15] have been extensively studied for their anti-oxidative properties. The regular intake of these has been associated to healthy life and potential for reducing the risk of chronic diseases.

This Special Issue (SI) of *Nutrients* sought high-quality research papers that delve into the intricate relationships between oxidative stress and human health, as well as the anti-oxidative effects of natural products derived from foods and herbal medicines. It also examines the underlying mechanisms that govern the protective actions of these products. The SI comprises 11 articles, including eight research papers and three reviews, authored by scholars hailing from diverse counties across the globe. These research endeavors encompass animal, cellular, and clinical studies, and cover neuroprotective, nephroprotective, liver damage amelioration, antioxidative, antimalarial, and anti-inflammatory effects.

Five articles revealed the oxidative stress-related mechanisms of the ingredients and extracts derived from herbal medicines or foods via in vivo and in vitro experiments. Jawad et al. [16] and Jeong et al. [17] highlighted the neuroprotective effects of N-methyl-(2S,4R)-Trans-4-hydroxy-L-proline derived from Sideroxylon obtusifolium and the ethanol extract of Vignae Radiatae Semem, respectively, on Aβ1–42-injected mouse models and neuronal HT22 and microglial BV2 cell lines. The mechanisms underlying these effects involved the alleviation of oxidative stress and a reduction in the generation of reactive oxygen species, respectively. Lam et al. [18] evaluated the effects of dihydro-resveratrol, which is distributed in plants of the Orchidaceae family and Cannabis sativa L., on insulin resistance, lipid accumulation and adipocyte differentiation in a high-fat-diet-induced mouse model; the primary mechanism comprised a reduction in the aggravation of oxidative stress via the modulation of the Nrf2-related antioxidative cascade. Wang et al. [19] revealed that Glabridin, an isoflavone extracted from Licorice root, alleviated ethanol-induced liver injury damage via a reduction in oxidative stress and inflammation through the p38 MAPK/Nrf2/NF-κB pathway. Aurori et al. [20] demonstrated that the nephrotoxicity induced by gentamicin can by mitigated via antioxidant therapy that utilizes an extract prepared from the fruits of Cornus mas and Sorbus aucuparia.

Two articles presented unique extraction techniques. Park et al. [21] employed a fermentation method to derive the antioxidative extract of Abelmoschus manihot using various Bacillaceae strains. Meanwhile, Stabrauskinene et al. [22] utilized cycodetrins to obtain a higher concentration of flavanones from grapefruits, resulting in increased antioxidant activities.

One clinical article [23] integrated research from the literature, machine learning, and genotyping techniques, along with colon assessments, to demonstrate the influence of genotype on the genotoxicity of processed red meat and the efficacy of protective phytochemical extracts when added to processed red meat.

Finally, three additional reviews [24,25,26] comprehensively summarized the impact of dietary polyphenols, lycopene, and anti-oxidative natural products on chronic diseases, antimalarial efforts, and orthodontic treatment.

All articles in this Special Issue shed new light on the utilization of anti-oxidative components or extracts derived from herbal medicines or foods in addressing oxidative stress-related diseases or issues related to food production; it also explicated the underlying mechanisms of their efficacy. As research continues to unravel the complexities of oxidative stress-related diseases and the mechanisms of action of natural products, there is growing optimism that these natural agents can be harnessed to develop innovative and effective treatments, contributing to the overall improvement of human health and well-being.
